# Explaining the Effects of Socioeconomic and Housing Characteristics on the Choice of Toilet Facilities among Ghanaian Households

**DOI:** 10.1155/2020/4036045

**Published:** 2020-05-20

**Authors:** William Adzawla, Hamdiyah Alhassan, Adams Imoru Jongare

**Affiliations:** ^1^University of Cheikh Anta Diop, West African Science Service Centre on Climate Change and Adapted Land Use (WASCAL), Climate Change Economics, Dakar, Senegal; ^2^University for Development Studies, Department of Agricultural and Resource Economics, Tamale, Ghana

## Abstract

Open defecation remains a major environmental sanitation challenge facing all areas of Ghana. This notwithstanding, the socioeconomic drivers of this phenomenon are overlooked. This study, therefore, analysed the factors that influence the choice of toilet facilities over the practice of open defecation in the country. Ghana Living Standard Survey round 7 (GLSS7) data were analysed using multinomial logit regression. From the data, a majority of households used improved toilet facilities (WC, KVIP, and pit latrines with slab) in Ghana and over one-fourth of households engaged in open defecation. The regression result revealed that the choice of toilet facilities over the practice of open defecation was significantly influenced by the sex of the household head, age, household size, education, marital status, locating in urban areas, regional locations, ownership of dwelling, type of dwelling, expenditure on rent, expenditure quintile, and per capita consumption expenditure of the household. Specifically, male, younger, less educated, and first income-quintile household heads have higher probability of practicing open defecation in Ghana. These variables point to specific policy directions that should be corrected or targeted to minimize, if not eliminate, the practice of open defecation in the country. The Media Coalition Campaign against Open Defecation should be intensified and directed towards the males, youths, and the less educated populace. This study also justified that calls for Ghanaians to change their attitudes or behavior towards open defecation are mere rhetoric if such calls are not defined within the socioeconomic conditions of the people of the area.

## 1. Introduction

There is a clear global commitment towards achieving an improved sanitation across the world. This is well captured by the world leaders' pledge under the Sustainable Development Goal 6 (SDG6). An important indicator under this goal is to “achieve access to adequate and equitable sanitation and hygiene for all and end open defecation” by the end of 2030 [1]. Cited in Weststrate et al. [2], the WHO/UNICEF (2016) defined improved sanitation as the use of “flush toilet, piped sewer system, septic tank, flush/pour flush to pit latrine, ventilated improved pit latrine (VIP), pit latrine with slab, composting toilet, and special case”. Global estimates show that access to improved sanitation is increasing although there are a significant number of persons with unimproved sanitation. Global open defecation decreased from 21% in 2000 to 9% (673 million) in 2017 [1]. Although this reduction points towards achieving SDG6, the numbers still remain high and worrisome. More importantly, there are regional disparities in the progress towards improved sanitation and ending open defecation. As such, while some regional areas have seen much improvement in access to improved sanitation facilities, others have made little progress. This may affect the overall global commitment as regional disparities may affect the global goal. Overall, the majority of people who lack access to basic sanitation services are found in the least developing countries and those living in the rural areas. Open defecation, for instance, is as high as 14% in sub-Saharan Africa (SSA) but only 2% in Latin America and the Caribbean regions [1]. The current rate of decline shows that over 5% of global population would still practice open defecation by 2030 [1]. The implication of these disparities is that the impact is most severe on the underserved. The institutional challenges to improved sanitation can be classified into five as collective action challenge, a coproduction challenge, a challenge of affordability versus acceptability, and a challenge related to housing tenure [3]. These challenges cannot be grossed over as Prüss-Ustün et al. [4] explained that inadequate drinking water, sanitation, and hygiene behaviours are major factors that influence global disease burden.

The effects of open defecation and poor sanitation continue to be a major sanitation theme being discussed among scholars and policy makers. Open defecation leads to the spread of disease, perpetuates under nutrition and poverty, and has a negative effect on personal dignity [3, 5–9] and slow pace of development [5]. Unfortunately, Ghana has missed out in achieving the MDG on basic sanitation [10]. Kumar and Sinha [5] explained that although open defecation continues to be a global norm, it poses the biggest health threat, especially to those living in the rural areas. The implication is that human capital deteriorates through health impacts if sanitation is unimproved. Environmental sanitation management was found to have implications for health, income, employment, productivity, and ecological sustainability [6]. Nonetheless, the merits of improved sanitation such as the use of improved toilet facilities and access to improved sanitation are low, especially in the rural areas [11]. As such, [11] recommended that in order for Ghana to achieve the SDGs, there is the need to strictly enforce sanitation laws of the country.

Ghana is classified under countries with 5–25% of the population engaging in open defecation, amidst inadequate availability of data [1]. Specifically, a total of 19% of Ghana's population practice open defecation [12] and this increased to 22% in 2017/18 assessment [13]. The practice of open defecation in the country varies, and this is high in the northern regions of Ghana and rural areas [10, 12–18]. Relatedly, 60% of the population live in communities where at least one household engages in open defecation [1]. The implication is that negative health effects of open defecation can have effect on 60% of the country's population. As at 2010, the economic cost of poor sanitation in Ghana is about 420 million Ghana cedis annually [19]. Of this, open defecation alone costs the country about 118 million Ghana cedis, an amount higher than that required to eliminate open defecation through the provisioning of latrines [19]. Unfortunately, the economic cost of open defecation is much higher for the poor than the rich. This complements other social and economic costs to prevent the poor from breaking their vicious poverty cycle. These observations suggest that more needs to be done in order to achieve the SDG target in the country. Expectedly, there have been several declared interests, “war” or “campaign” against open defecation in the country and these are generally led by the media. In 2018, for instance, a group known as “Media Coalition against Open Defecation” was lunched with the aim of improving public sensitization on open defecation through community outreach programmes as well as challenging the institutions connected to eliminating open defecation to improve their efforts [15].

The choice of toilet facility can be determined by a number of factors. According to Appiah-Effah et al. [10], the status of Ghana's sanitation is influenced by economic, institutional, and socioeconomic factors. Hence, open defecation in Ghana can be attributed to a number of factors including behavior and attitude, cultural, poverty, and socioeconomic barriers. For instance, Osumanu and Kosoe [8] outlined that people engage in open defecation due to financial constraints and the lack of private and public toilet facilities in the country. However, public toilets in the country are not only inadequate but also have poor hygienic conditions that drive people away from its usage and preference for open defecation [8]. Although this study provided some information related to open defecation, it is limited to only one city, Wa of Ghana. Similarly, despites the optimism expressed by Appiah-Effah et al. [10] on recent sanitation reforms in the county, it cannot be said that Ghana's socioeconomic research has provided adequate empirical evidence on the role of socioeconomic and housing characteristics on open defecation in the country. As such, much of the discussion on open defecation was left to politicians, civil societies, and the media. These do not show any good signal for understanding the role of people's characteristics on open defecation in the country. Even if there are more research studies on the subject that were not accessed by the researchers, it is appropriate that periodic research on the subject is done. To address this shortfall and provide policy information on how to address open defecation in the country, this study is set to analyse the effects of the socioeconomic, location, housing, and income inequality characteristics on open defecation in Ghana. With the emergence and emphasis on community-let total sanitation where communities are made to understand the impacts of poor sanitation and to trigger them take actions [14], the role of socioeconomic and housing characteristics of households become necessary.

## 2. Methodology

### 2.1. Study Location

The study was conducted in Ghana. Ghana is a tropical country located on the west coast of Africa with a 2019 population of about 30,093,201 and a 2018 economic growth rate of 5.6% [20]. Largely, poverty levels in the country are declining from about 31.9% in 2005/6 to 24.2% in 2012/13 and 23.4% in 2016/17 [18]. Nonetheless, inequality between males versus females, urban versus rural areas, and regional differences continues to remain significant. Overall, inequality, measured by Gini coefficient, of Ghana has marginally increased from 41.9% in 2005/6 to 42.3% in 2012/13 and 43% in 2016/17 [18]. The level of inequality is high in rural areas (41.8%) than urban areas (37.9%) [18]. While 5% of the population are located in the first quintile, 47.9% are located in the fifth quintile [21].

Nonetheless, Ghana's classification as a lower middle-income country in SSA, open defecation remains a major sanitation challenge for the country. For instance, in 2016/17, 5.9% and 29% of urban and rural households, respectively, had no toilet facility and therefore practice open defecation in bushes, beaches, and fields. While as high as 77.1% of households in the Upper East Region practice open defecation, as low as 4% of households in the Greater Accra Region practice open defecation [18]. Such open defecation, especially in beaches, reduces the ecotourism values associated with beaches and the coastal areas of the country.

Ghana's sanitation management is regulated by the Environmental and Sanitation Policy whose primary goal is to develop “clear and nationally accepted vision of environmental sanitation as an essential social service and a major determinant for improving health and standard of living in Ghana” [7]. In this policy, there are assigned responsibilities to both individuals, communities, and local authorities such as District Assemblies on sanitation management. For instance, the District Assemblies are required to ensure the availability of facilities for the disposal of liquid waste in their districts. Generally, the policy recommended the use of various toilet facilities including water closet (WC) and septic tank system, pour flush latrine, and ventilated improved pit latrines in Ghana [7].

### 2.2. Data Type and Analysis

This study relied on GLSS7 data. These are national data that seek to comprehensively assess Ghanaian households and their characteristics. The data were downloaded from the Ghana Statistical Service (GSS) website upon expression of interest by the authors to understand the sanitation conditions in the country. From the data, a total of 14,154 households were used for this study. Information on the toilet facilities used by the households was categorized from the original data for the convenience of this study. The data sorted were analysed through a multinomial logit regression in STATA 14.

Multinomial logit regression is an econometric method used in analysing nominal data where there are no meanings to the values assigned to the variable labels (Greene, 2003). For instance, in this study, open defecation is assigned 0 while WC was assigned 3. However, these assigned values (0 and 3) are meaningless in their own rights but to enable the software to read the data and allow estimation. Thus, we could have assigned any positive integer, say, 100 to open defecation and 1 to WC with no effect on the result. Primarily, the multinomial logit regression assumes that if households are faced with several toilet facilities, the household uses only one alternative. Therefore, households in this study are assumed to be using solely their reported toilet facilities and this allowed the study to proceed with the formulation of a multinomial logit regression to fit the data set. In this study, open defecation is used as a reference group to all other toilet facilities (public toilet, pit latrine/KVIP, and WC). The analytical procedure of the multinomial logit is described as follows.

Given that an individual *i* receives a utility from choosing a particular toilet facility *j*, then this can be defined as

(1) $$\begin{array}{c} U_{ij}=\beta^{\prime }X_{ij}+\varepsilon_{ij}, \end{array}$$

where *U*_*ij*_ is the derived utility, *β* is a vector of parameters, *X* is a vector of independent variables, and *ε* is an error term. Assuming there are *J* alternatives, the probability of choosing alternative *k* is given as

(2) $$\begin{array}{c} \mathrm{Pr}\left(y=k\right)=\mathrm{Pr}\left(U_{k}>U_{j}\text{for all}j=k\right). \end{array}$$

Redefining the probability of choice from equation (1),

(3) $$\begin{array}{c} {\mathrm{Pr}}_{ij}=\frac{e^{X_{i}^{\prime }\beta_{j}}}{\sum_{i=1}^{J}e^{X_{i}^{\prime }\beta_{j}}};\;j=1,\ldots ,J. \end{array}$$

Therefore, if *β*(1) is assigned 0, then the set of coefficients estimated in this study can be defined as

(4) $$\begin{array}{c} {\mathrm{Pr}}_{\left(y=1\right)}=\frac{1}{1+e^{X\beta \left(2\right)}+e^{X\beta \left(3\right)}},\\ {\mathrm{Pr}}_{\left(y=1\right)}=\frac{e^{X\beta \left(2\right)}}{e^{X\beta \left(2\right)}+e^{X\beta \left(3\right)}},\\ {\mathrm{Pr}}_{\left(y=1\right)}=\frac{e^{X\beta \left(3\right)}}{e^{X\beta \left(2\right)}+e^{X\beta \left(3\right)}}. \end{array}$$

Given this formulation, the empirical model estimated is given as

(5) $$\begin{array}{c} \text{Toilet facility}=\beta_{1}\text{Sex}+\beta_{2}\text{Age}+\beta_{3}\text{Education}+\beta_{4}\text{Household size}+\beta_{5}\text{Marital status}+\beta_{6}\text{Urban}+\beta_{7}\text{Region}\\ +\beta_{8}\text{Ownership of delling}+\beta_{9}\text{Dwelling type}+\beta_{10}\text{Rent expenditure}+\beta_{11}\text{Welfare quintlie}+\beta_{12}\text{Per capita expenditure}, \end{array}$$

where the definitions of the variables are provided in Table 1.

Table 1 shows that the average household head's age was 46.3 years with an educational level of 3 years. The data shows that about 63% of the household heads had no formal education. The average household size from the sample was about 4, and this is consistent with a mean household size of about 4 in 2010 population and housing census of Ghana [16]. The average imputed rent on housing was GHS685.10 per annum. This suggests a largely low imputed rent, perhaps due to undervaluation of rents from personal houses. Averagely, the per capita expenditure of the households was GHS3,606.50, far higher than the upper poverty line of GHS1,314.00 and the average welfare of GHS2,431.43 in Ghana [17]. This suggests that, granted the poverty line remained unchanged, the average household head can be considered as nonpoor. About 68% of the households are headed by males while about 63% of the household heads are currently married. About 57% of the selected household heads are located in the rural areas, and the majority (12.3%) are found in the Ashanti region of Ghana. The majority of the household heads are located in either the homes of family relations or personal homes. The major type of dwelling used by the households is compound houses. These compound houses are mostly rented houses where several families are located in the same house. While about 24% of the household heads have higher welfare levels and classified in the fifth welfare quintile, as high as 22% are also located in the first welfare quintile.

## 3. Results and Discussions

### 3.1. Toilet Facilities Used by Ghanaian Residents

Table 2 shows the percentage distribution of the various toilet facilities and the practice of open defecation by Ghanaian households located in rural and urban areas. Using the UNICEF and WHO's [22] classification of sanitation or toilet facilities, the result shows that the highest percentage (44.9%) of the households used improved toilet facilities in the county. This improved sanitation or toilet facilities include pit latrines with slab (19.4%), KVIPs (12.1%), and WC (13.4%). Shared toilet facility that involves the use of public toilets is the second most used toilet facility in the country while 0.4% of households used unimproved toilet facilities such as buckets and pans. The public toilets include KVIPs, pit latrines, or WCs that are established by private or public agencies and used by the public for a fee. The remaining 27.6% of the households engaged in open defecation in the country; thus, over one-fourth of the households defecate in the open. Considering the observed difference in WC and KVIP/pit latrines in the country, this study considered these two improved practices as separate facilities in the econometric estimation while households who used unimproved facilities were dropped due to its small proportion.

Geographically, open defecation and the use of pit latrines and KVIP were common among rural households while the use of WC and public toilets were common among urban households. The high open defecation in rural areas than urban areas is consistent with earlier reports by Appiah-Effah et al. [10, 12–18, 21]. For [3], the fact that the level of use of improved sanitation facilities is higher in the urban areas than the rural areas should not be jubilated because progress has been slow in the urban areas. Similarly, the spread of negative outcomes such as disease from poor sanitation is severe in the urban areas since the human densities are high. The high open defecation in the country is a major sanitation challenge that poses environmental and health threats to the general Ghanaian public. This justifies media pronunciation for ending open defecation in the country. The level of open defecation is higher than the average 14% open defecation in SSA [1]. Nonetheless, this may not be an isolated finding for Ghana since [23] found that 23.2% of the households practice open air defecation in rural villages of the Raipur district of India.

In addition to Table 2, the choice of toilet facilities and open defecation by region is provided in Table 3. This shows that open defecation is significantly common in Upper East (77.5%), Northern (62.9%), and Upper West (56.4%) regions of Ghana and lower in Greater Accra Region (5.6%), Ashanti Region (6.2%), and Eastern Region (6.8%). On the other hand, the use of WC was highest for households in the Greater Accra Region and lowest for households in the Northern Region.

### 3.2. Factors Influencing the Choice of a Toilet Facility

Table 4 shows the result of the multinomial logit regression on the factors that influenced the choice of toilet facilities by Ghanaian households. As indicated under data analysis, the reference group is open defecation; therefore, the discussions are done for each toilet facility in relation to the practice of open defecation. The result shows that the practice of open defecation over the choice of various toilet facilities was influenced by the sex of the household head, the age, household size, education, marital status, locating in urban areas, regional locations, ownership of dwelling, type of dwelling, expenditure on rent, expenditure quintile, and per capita consumption expenditure of the household. The implication of each of these significant factors is discussed subsequently.

The variable sex had a negative effect on the choice of toilet facilities over the practice of open defecation, and this is significant for only public toilet and WC facilities. Relative to open defecation, the marginal effects show that the male heads have, respectively, 0.027 and 0.007 probabilities less of using public toilets and WC toilets than female heads. This result is conceivable since female heads and females in general often feel shy in defecating in the open than males. Studies also show that women who engage in open defecation are more vulnerable to infections and nonpartner sexual violence than men [4, 23–25]. Using a chi-square test, Kumar and Sinha [5] and Panda et al. [23] found that men practice open defecation than women. Contrarily, Akpakli et al. [11] found that male heads are more likely to use improved sanitation facilities compared to female heads.

Age had a positive effect on the choice of toilet facilities over the practice of open defecation, and this is significant for public and WC toilet facilities. The marginal effects were all 0.001. The implication is that the higher the age of the household head, the higher the probability of using public or WC toilet over open defecation. In most Ghanaian settings, age comes with high social responsibility and the behavior of the elderly provides moral ground to advice the youths against open defecation. In order for the elderly to maintain their social status in their communities, they may not like to engage in open defecation. This result is also consistent with general observation where younger persons are mostly seen engage in open defecation. Relatedly, the elderly household heads might have accumulated enough capital over time to either invest in the construction of personal toilet facilities or pay for the use of public toilet facilities on a daily basis. Consistent with this study, Akpakli et al. [11] found that household heads with 40–69 years are less likely to practice open defecation than those with ages below 31 years and argued that the majority of those in higher age groups might be economically active and, hence, able to afford toilet facilities.

The effect of education on the choice of toilet facilities by household heads is positive and significant for pit latrines/KVIP and WC. Thus, the highly educated household heads have higher probabilities of using pit latrines/KVIP and WC and do not practice open defecation. This is because, not only does education improves the understanding of a person on the health implications of open defecation but also the literate in Ghanaian societies are more under public criticism for wrongful attitudes. A common description of literates who engage in socially or morally unfit acts is “ (s)he not the one who completed school” or “I thought his/her education should make him/her better.” These are damming comments which denigrate the value of one's educational status and can be tired to Kumar and Sinha [5] explanation that open defecation has a negative consequence on personal dignity. As a consequence, the highly educated household heads would not want to engage in open defecation. Economically, the educated household heads might be engaged in high-income economic activities and would, therefore, be able to pay for the construction of toilet facilities or their usage. This result is consistent with those of Kumar and Sinha [5], Osumanu et al. [9], Akpakli et al. [11], Abubakar [26], and Panda et al. [23].

Household size is significant in explaining the choice of toilet facilities by Ghanaian households. While heads with larger household size have higher probability of choosing public toilets over open defecation, heads with lesser household size have higher probability of practicing open defecation over the use of pit latrines/KVIP and WC. Since almost every household member would prefer going to toilets in the morning before going out or in the evening before sleep, it requires that more toilet facilities are required at home; otherwise, some household members would go to the public toilets or in the open where there is little to zero waiting time. Osumanu et al. [9] also estimated a similar result where the respondents indicated that the cost of building household toilet facilities is high and hence not a necessary investment for the family.

Current marital status of household heads had a positive significant effect on the probability of using pit latrines/KVIP and WC over the practice of open defecation. This suggests that household heads who are currently married have higher probabilities of using the former toilet facilities over the latter. This is because couples often would want to have high level of privacy in almost every aspect of their lives. Therefore, it is expected that they would prefer private toilet facilities such as pit latrines than going to the open or public toilet. A similar result was obtained by Akpakli et al. [11] in Ghana.

Two location variables are considered in this study: the urban or rural areas and regional locations. All these factors had significant effect on the choice or use of at least one toilet facility over the practice of open defecation. The result suggests that household heads located in urban areas have higher probability of using public toilets and WC over the practice of open defecation but a lower probability of using pit latrines/KVIP over open defecation. Generally, while WC and public toilet facilities are common in the urban areas (refer to Table 2), the pit latrines/KVIP is common in the rural areas. Therefore, this finding can be attributed to the availability of the various toilet facilities. For instance, the use of WC is a feature of urbanization. The effect of regional location on the choice of toilet facilities is mix. However, it is generally observed that households located in the Greater Accra Region have higher probability of using public toilet facilities and do not engage in open defecation. This suggests that the high focus of open defecation campaigns in Accra has to be reassessed by all policy makers including the media, and equal if not more emphasis should be placed on the major cities of the other regions. Except Northern and Upper East regions, households located in the Greater Accra Region have higher probabilities of using WC over open defecation. This was expected due to the high availability of WC facilities in Accra and its environs such as Tema than in the major cities of the other regions. Again, except Upper East and Northern regions, households in all other regions other than in the Greater Accra Region have higher probabilities of using pit latrines/KVIP over open defecation. These results are consistent with the observations made in Table 2 and also confirmed the results of Abubakar [26] and Akpakli et al. [11].

The three housing characteristics considered under this study all had significant effect on the usage of a particular toilet facility. The result on the ownership of dwelling showed that household heads that are located in houses that are not personally owned or owned by family relative have higher probabilities of using public toilet and WC over the practice of open defecation but have higher probability of practicing open defecation over the use of pit latrines/KVIP. On WC, most government and private residents such as estates have WC provisions in the design of the houses. Also, one condition people use in accepting accommodations for rent from private entities is the availability of toilet facilities. Therefore, based on the economic or income status of a person, people always prefer where there is a WC or a nearby decent public toilet. The result also shows that household heads that resides in houses such as compound, flats, or semidetached houses have significantly higher probabilities of using pit latrines/KVIP over practicing open defecation. Similarly, those residing in flats or semidetached houses have significantly higher probabilities of using WC over open defecation. This result suggests that persons who have no decent accommodation such as perching or slums have higher probability of practicing open defecation. Consistently, the result established that the higher the rent on housing, the higher the probability of using pit latrines/KVIP and WC but the lesser the probability of using public toilets over the practice of open defecation. This is because the cost of housing is based on its characteristic such as the presence of a toilet facility. The improved the characteristics, the higher the rent. It is therefore not surprising that household heads who paid higher rents would be unwilling to go to public toilets that are often unhygienic but rather practice open defecation. Evidently, Osumanu and Kosoe [8] reported that public toilets in the WA municipality, for instance, do not meet the local sanitation needs of the people, hence creating more problems and pushing residents into open defecation. In a related study, Abubakar [26] found that housing characteristics such as the roof material and number of rooms have significant influence on open defecation in Nigeria. Similarly, housing tenure challenges reduce the household's incentive to invest in improved toilet facilities; for instance, the fear of eviction from a rented home or insecure rental tenure can affect the decision to invest in sanitary improvement [3].

Welfare quintile had significant effects in explaining the types of toilet facilities used by Ghanaian households. Overall, households located in the second, third, fourth, and fifth quintiles significantly have higher probabilities of using WC and public toilets as against the practice of open defecation. This suggests that the poor-class households (the first quintile households) often engage in open defecation than the rich- and the middle-class households. This is because the primary focus of the poor or those in the first quintile is how to provide food to their families and not how to deal with waste (a by-product from eating). Consistently, McGranahan [3] described that poor persons who depend on very low incomes cannot be expected to afford improved sanitation as their main focus is on the provisioning of basic needs such as food and clothing. On the contrary, the result shows that household heads in the second, third, and fourth quintiles have lesser probability of using pit latrines/KVIP over the practice of open defecation than those in the first quintile. There is also nearly negligible but significant marginal effect that suggests that an increase in the actual per capita expenditure decreases the choice of WC over the practice of open defecation. Panda et al. [23] also found that there is a significant association between the socioeconomic status (poor, middle, or rich) and open defecation where the poor-class households practice open defecation more in India. In Ghana, Akpakli et al. [11] also found that the poor have less chance of using improved sanitation facilities since the rich have a major role in the acquisition and utilization of improved sanitation facilities such as toilets. Consistently, Osumanu et al. [9] estimated that an increase in income decreases the probability of open defecation in the Wa Municipality of Ghana.

## 4. Conclusions and Recommendations

Open defecation remains a major sanitation challenge in Ghana and over the years, it is one of the topical sanitation issues in the Ghanaian media. Therefore, this study outlined the socioeconomic factors that influenced open defecation in the country. This study was articulated on the basis that these socioeconomic factors drive the expressed behavior or attitude of people to engage in open defecation and, hence, should be considered as fundamental factors to tackle and not blame open defecation solely on “behavioural attitudes”. Evidently, this study established that open defecation is higher in the country. Specifically, more than one in every five households engaged in open defecation in the country. This observed level of open defecation in Ghana far exceeds the average in SSA, thereby raising concern for the country.

The study established that several socioeconomic and housing characteristics significantly explained the choice of the toilet facility used by the households. These includes the sex of the household head, the age, household size, education, marital status, locating in urban areas, regional locations, ownership of dwelling, type of dwelling, expenditure on rent, expenditure quintile, and per capita consumption expenditure of the household. It is concluded, therefore, that open defecation is common among the male, younger, less educated, and household heads in the first income quintile. The housing characteristics also indicated that the provision of private and government residents is appropriate in addressing open defecation challenges in the country. Largely, the findings can be tired to the economic status of the households, as the poor or as it may be related to other factors have the tendency towards the practice of open defecation. It is, therefore, concluded that any call for Ghanaians to change their attitudes or behaviours towards open defecation should be rooted in the socioeconomic and housing conditions of the people of the area; otherwise, such calls may be mere rhetoric. As a consequence, government should take steps to ensure that house owners provide toilet facilities in their homes. This can be achieved through two major ways. First, to offset the positive effect of low income and housing characteristics on open defecation, affordable housing projects by successive governments have to be taken more seriously and implemented to its fullest. This is because such housing policies come along with sanitation management plans. Secondly, community-led total sanitation should be promoted among communities to help the community members pull resources together for the construction of toilet facilities either at household level or at specific community locations. This, for instance, can reduce open defecation among the males and the youths, as they would be deeply engaged in such community commitments. Government through the Ministry of Local Government must take interest in ensuring that public toilet facilities in the country are kept clean and conducive for use. This would demotivate people, especially the literates from the use of open defecation across the country.

## Figures and Tables

**Table 1:** Definition of variables.

Variable	Description	Mean^a^ (%)
Age	Years from birth	46.3^a^
Education	Total number of years of formal education	3.0^a^
Household size	Number of people in the same home and eating from the same pot	4.2^a^
Rent expenditure	The total imputed rent cost for all housing in Ghana cedis	685.1^a^
Per capita expenditure	The total annual expenditure in Ghana cedis per an individual of a household	3,606.5^a^
Sex	Males (1)	68.7
	Females (0)	31.3
Marital status	Married (1)	63.9
	Single (0)	36.1
Urban	Urban (1)	42.8
	Rural (0)	57.2
Region	Greater Accra (0)	9.9
	Ashanti (1)	12.3
	Brong Ahafo (2)	9.4
	Central (3)	9.4
	Eastern (4)	10.1
	Northern (5)	10.1
	Upper East (6)	9.7
	Upper West (7)	9.7
	Volta (8)	10.0
	Western (9)	9.5
Ownership of dwelling	Own (relative/purchased) (0)	51.9
	Private entity/individual (1)	20.8
	Government (2)	27.3
Dwelling type	Others	1.3
	Compound	62.6
	Flat/semidetached/separate house	36.1
Welfare quintile	First (0)	22.3
	Second (1)	18.0
	Third (2)	17.0
	Fourth (3)	18.6
	Fifth (4)	24.1

Reference groups in the categorical variables are given a value of “0”; values with superscript a are means. The assumption is that the level of open defecation would be high in the reference groups.

**Table 2:** Distribution of toilet facilities and open defecation by locality.

UNICEF/WHO classification	GLSS classification	Urban		Rural		Total	
		Freq.	%	Freq.	%	Freq.	%
Open defecation	Open defecation	565	9.3	3,343	41.3	3,908	27.6

Improved	WC	1,656	27.4	242	3.0	1,898	13.4
	KVIP	944	15.6	766	9.5	1,710	12.1
	Pit latrine	704	11.6	2,041	25.2	2,745	19.4

Unimproved	Bucket/pan	23	0.4	34	0.4	57	0.4
Shared	Public toilet	2,161	35.7	1,675	20.7	3,836	27.1

Total		6,053	100.0	8,101	100.0	14,154	100.0

**Table 3:** Distribution of toilet facilities and open defecation of regions in Ghana.

Region	Open defecation	Improved			Unimproved	Shared	Total frequency
	Open defecation (%)	WC (%)	Pit latrine (%)	KVIP (%)	Bucket/pan (%)	Public toilets (%)	
Western	10.8	17.2	30.4	11.5	0.2	29.8	1337
Central	15.1	11.1	17.6	24.7	0.1	31.3	1327
Greater Accra	5.6	37.7	8.1	13.0	0.1	35.5	1401
Volta	24.3	7.0	27.2	13.2	0.1	28.3	1420
Eastern	6.8	10.5	33.3	21.6	0.3	27.4	1432
Ashanti	6.2	27.7	19.0	9.0	1.0	37.1	1735
Brong Ahafo	14.9	9.1	21.7	11.9	0.3	42.2	1330
Northern	62.9	2.4	7.4	4.9	0.5	21.9	1427
Upper East	77.5	3.3	9.4	4.6	0.1	5.0	1375
Upper West	56.4	4.6	20.1	7.4	1.2	10.4	1370
Total	27.6	13.4	19.4	12.1	0.4	27.1	14154

Percentages were calculated over regional total frequency; source, GLSS7 data.

**Table 4:** Factors influencing the usage of other toilet facilities over open defecation.

Variable	Public toilet			Pit latrine/KVIP			WC		
	Coef. [std. err.]	*Z* value (*P* value)	mfx	Coef. [std. err.]	*Z* value (*P* value)	mfx	Coef. [std. err.]	Z value (*P* value)	mfx
Sex	−0.161 [0.075]	−2.150 (0.032)	−0.027^*∗∗*^	−0.026 [0.072]	−0.370 (0.713)	0.020	−0.206 [0.098]	−2.110 (0.035)	−0.007^*∗*^
Age	0.011 [0.002]	5.580 (0.000)	0.001^*∗∗*^	0.010 [0.002]	5.600 (0.000)	0.001	0.021 [0.003]	7.350 (0.000)	0.001^*∗∗∗*^
Education	0.059 [0.008]	7.180 (0.000)	0.002	0.067 [0.008]	8.410 (0.000)	0.006^*∗∗∗*^	0.113 [0.010]	11.670 (0.000)	0.003^*∗∗∗*^
Household size	0.023 [0.013]	1.670 (0.095)	0.007^*∗∗∗*^	−0.012 [0.012]	−1.000 (0.317)	−0.004^*∗*^	−0.074 [0.021]	−3.520 (0.000)	−0.004^*∗∗∗*^
Marital status	0.202 [0.076]	2.640 (0.008)	−0.002	0.272 [0.073]	3.740 (0.000)	0.029^*∗∗*^	0.481 [0.101]	4.780 (0.000)	0.014^*∗∗∗*^
Urban	1.472 [0.072]	20.500 (0.000)	0.156^*∗∗∗*^	0.850 [0.071]	12.020 (0.000)	−0.045^*∗∗∗*^	[0.104]	24.330 (0.000)	0.080^*∗∗∗*^

*Region (Accra)*									
Ashanti	0.615 [0.172]	3.580 (0.000)	−0.047^*∗∗*^	0.921 [0.175]	5.260 (0.000)	0.076^*∗∗∗*^	1.509 [0.187]	8.090 (0.000)	0.056^*∗∗∗*^
Brong Ahafo	0.200 [0.163]	1.230 (0.219)	−0.030	0.451 [0.166]	2.720 (0.007)	0.070^*∗∗∗*^	0.266 [0.198]	1.350 (0.179)	−0.0003
Central	−0.331 [0.162]	−2.050 (0.041)	−0.139^*∗∗∗*^	0.429 [0.163]	2.640 (0.008)	0.161^*∗∗∗*^	−0.263 [0.191]	−1.370 (0.170)	−0.013^*∗∗∗*^
Eastern	0.430 [0.180]	2.390 (0.017)	−0.183^*∗∗∗*^	1.586 [0.178]	8.890 (0.000)	0.297^*∗∗∗*^	0.698 [0.206]	3.380 (0.001)	−0.012^^*∗∗*^^
Northern	−1.555 [0.157]	−9.910 (0.000)	−0.148^*∗∗∗*^	−1.783 [0.165]	−10.790 (0.000)	−0.172^*∗∗∗*^	−1.803 [0.256]	−7.050 (0.000)	−0.023^*∗∗∗*^
Upper East	−3.200 [0.188]	−17.010 (0.000)	−0.357^*∗∗∗*^	−1.892 [0.163]	−11.580 (0.000)	−0.136^*∗∗∗*^	−1.948 [0.235]	−8.300 (0.000)	−0.019^*∗∗∗*^
Upper West	−1.785 [0.171]	−10.450 (0.000)	−0.268^*∗∗∗*^	−0.698 [0.160]	−4.380 (0.000)	0.040	−0.373 [0.222]	−1.680 (0.093)	0.026^*∗∗*^
Volta	−0.608 [0.158]	−3.850 (0.000)	−0.138^*∗∗∗*^	0.066 [0.158]	0.420 (0.676)	0.118^*∗∗∗*^	−0.768 [0.200]	−3.840 (0.000)	−0.020^*∗∗∗*^
Western	0.274 [0.170]	1.610 (0.107)	−0.107^*∗∗∗*^	0.908 [0.170]	5.320 (0.000)	0.149^*∗∗∗*^	1.030 [0.194]	5.310 (0.000)	0.028^*∗∗∗*^

*Ownership of dwelling*									
Private entities	0.653 [0.096]	6.800 (0.000)	0.076^*∗∗∗*^	0.313 [0.095]	3.300 (0.001)	−0.04^*∗∗∗*^	1.170 [0.124]	9.430 (0.000)	0.041^*∗∗∗*^
Government	0.404 [0.072]	5.570 (0.000)	0.056^*∗∗∗*^	0.108 [0.069]	1.550 (0.120)	−0.047^*∗∗∗*^	0.907 [0.106]	8.520 (0.000)	0.036^*∗∗∗*^

*Dwelling type*									
Compound	0.608 [0.246]	2.470 (0.013)	0.025	0.823 [0.263]	3.130 (0.002	0.116^*∗∗∗*^	0.650 [0.325]	2.000 (0.045)	0.004
Flats/semidetached/separate house	0.725 [0.250]	2.900 (0.004)	0.002	1.026 [0.266]	3.860 (0.000)	0.119^*∗∗∗*^	1.825 [0.329]	5.550 (0.000)	0.063^*∗∗∗*^
Expenditure of rent	−0.0006 [0.0001]	−6.660 (0.000)	−0.0002^*∗∗∗*^	0.000 [0.000]	4.370 (0.000)	0.0001^*∗∗∗*^	0.001 [0.000]	14.780 (0.000)	0.00005^*∗∗∗*^

*Welfare quintile*									
Second	0.410 [0.089]	4.610 (0.000)	0.075^*∗∗∗*^	−0.028 [0.077]	−0.360 (0.720)	−0.065^*∗∗∗*^	1.028 [0.272]	3.780 (0.000)	0.023^*∗∗∗*^
Third	0.693 [0.101]	6.890 (0.000)	0.099^*∗∗∗*^	[0.091]	1.990 (0.047)	−0.065^*∗∗∗*^	1.724 [0.266]	6.470 (0.000)	0.046^*∗∗∗*^
Fourth	0.848 [0.119]	7.120 (0.000)	0.090^*∗∗∗*^	0.414 [0.111]	3.730 (0.000)	−0.043^*∗∗*^	2.194 [0.270]	8.120 (0.000)	0.067^*∗∗∗*^
Fifth	1.215 [0.182]	6.660 (0.000)	0.096^*∗∗∗*^	0.813 [0.177]	4.590 (0.000)	−0.021	2.801 [0.305]	9.190 (0.000)	0.091^*∗∗∗*^
Per capita consumption expenditure	1.6 *E* − 05 [2.2 *E* − 05]	7.4 *E* − 01 (4.6 *E* − 01)	0.000001	1.1 *E* − 05 [2.2 *E* − 05]	4.8 *E* − 01 (0.000)	−0.000001	7.0 *E* − 05 [2.2 *E* − 05]	3.1 *E* + 00 (0.000)	0.000003^*∗∗∗*^
Constant	−1.7 *E* + 00 [3.0 *E* − 01]	−5.5 *E* + 00 (0.000)		−1.8 *E* + 00 [3.1 *E* − 01]	−5.9 *E* + 00 (0.000)		−7.5 *E* + 00 [4.6 *E* − 01]	−1.6 *E* + 01 (0.000)	

## Data Availability

Data will be made available upon request.
